# Contraceptive preferences and adoption following female genital fistula surgery in Uganda: a mixed-methods study

**DOI:** 10.1186/s12978-023-01663-3

**Published:** 2023-08-19

**Authors:** Alison M. El Ayadi, Hadija Nalubwama, Caitlyn Painter, Othman Kakaire, Suellen Miller, Justus Barageine, Josaphat Byamugisha, Susan Obore, Abner Korn, Cynthia C. Harper

**Affiliations:** 1 Department of Obstetrics, Gynecology and Reproductive Sciences, University of California San Francisco, 550 16th Street, 3rd Floor, San Francisco, CA 94518 USA; 2 Department of Epidemiology and Biostatistics, University of California San Francisco, San Francisco, USA; 3 Department of Obstetrics and Gynaecology, Makerere University College of Health Sciences, Kampala, Uganda; 4 Urogynaecology Division, Mulago National Teaching and Referral Hospital, Kampala, Uganda

**Keywords:** Female genital fistula, Surgery, Contraception, Family planning, Fertility intention

## Abstract

**Background:**

Female genital fistula, largely caused by prolonged obstructed labour, is treated by surgical repair. Preventing pregnancy for a minimum period post-repair is recommended to ensure adequate healing and optimize post-repair outcomes.

**Methods:**

We examined contraceptive preferences and use among Ugandan fistula patients (n = 60) in the year following genital fistula surgery using mixed-methods. Sexual activity, contraceptive use and pregnancy status were recorded quarterly for 12 months after surgery. In-depth interviews among purposively selected participants (n = 30) explored intimate relationships, sexual experiences, reproductive intentions, and contraceptive use.

**Results:**

Median participant age was 28 years and almost half (48%) were married or living with partners. Counselling was provided to 97% of participants on delaying sexual intercourse, but only 59% received counselling on contraception. Sexual activity was reported by 32% after 6 months and 50% after 12 months. Eighty-three percent reported not trying for pregnancy. Among sexually active participants, contraceptive use was low at baseline (36%) but increased to 73% at 12 months. Six (10%) women became pregnant including two within 3 months post-repair. Interview participants who desired children immediately were young, had fewer children, experienced stillbirth at fistula development, and felt pressure from partners. Women adopted contraception to fully recover from fistula surgery and avoid adverse outcomes. Others simply preferred to delay childbearing. Reasons cited for not adopting contraception included misconceptions related to their fertility and to contraceptive methods and insufficient or unclear healthcare provider advice on their preferred methods.

**Conclusions:**

A high proportion of patients were not counselled by healthcare providers on contraception. Provision of comprehensive patient-centred contraceptive counselling at the time of fistula surgery and beyond is important for patients to optimize healing from fistula and minimize recurrence, as well as to meet their own reproductive preferences.

## Abstract (plain language summary)

Female genital fistula is an injury that can happen during childbirth if women do not get high-quality care. It can be treated by surgery. After fistula surgery, women should not get pregnant right away so they can heal. To help women heal, we need to know how women with fistula think and feel about birth control. We collected data from 60 Ugandan women for one year after fistula surgery to learn about sex after surgery, contraceptive use, and pregnancy. We talked with 30 of them to learn more about their relationships, experiences with sex, plans for children, and birth control use. Most women were told to not have sex right away (97%) and many were told about birth control (59%). Some women had sex by 6-months after surgery (32%) and half by 12-months (50%). Most did not want to get pregnant (83%). Contraceptive use rose to 73% among those having sex by 12-months. Women who wanted to get pregnant soon were young, had fewer children, had lost their baby at fistula, and their partners wanted a child. Women told us they used contraception so they could recover from surgery or because they did not want a child right away. Some women told us they did not use contraception because they were worried it would hurt them. Others did not know enough about contraception. Patient education on contraception is needed at fistula surgery to help patients heal and meet their plans for children.

## Introduction

Female genital fistula in low- and middle-income countries is primarily caused by prolonged obstructed labour in the absence of high-quality emergency obstetric care, resulting in significant maternal morbidities. Fistula may also arise from iatrogenic or traumatic aetiologies. The primary presenting symptom is uncontrolled urine and/or faecal incontinence, depending on the fistula location, and may also be associated with a wider range of injuries broadly affecting gynaecological, urological, gastrointestinal, neurological, and/or musculoskeletal systems [1]. Prevalence and incidence data are limited; however, between 500,000 to 2 million women worldwide are estimated to live with the condition [2, 3], with an annual incidence of 50,000–100,000 [4]. The majority of cases occur in sub-Saharan Africa, including in Uganda [5]. Women with fistula experience significant physical, psychosocial and economic morbidity [6]. Surgical correction is the cornerstone of treatment for women with fistula, with fistula closure rates as high as 94% [7].

Many women with fistula also have experienced stillbirth, and with generally high fertility desires in low-income settings where fistula occur, some women who undergo fistula repair express an intent for subsequent childbearing. Although no formal guideline exists regarding the minimum amount of time that women should wait before becoming pregnant following fistula repair, providers often recommend waiting until fully recovered from surgery, typically somewhere between 3 to 12 months, to reduce the risk of fistula repair breakdown and to optimize pregnancy outcomes [8, 9]. For example, EngenderHealth’s FistulaCare and FistulaCarePlus programs, two large USAID-funded programs on fistula, recommended 3–6 months of abstinence from sexual intercourse, followed by an additional period of contraception prior to post-repair pregnancy [14].

Given the importance of delaying birth following fistula repair, contraceptive counselling is an essential component of health education for both patients and their partners in the perioperative period. The few studies that have focused on contraception following fistula repair have found low uptake, ranging from 20% to 37% in Kenya [10], Malawi [11], Nigeria [12–14], and the Democratic Republic of the Congo [15]. Available research suggests that non-adoption of contraception following fistula repair is due to socioeconomic reasons, religious and cultural beliefs, and myths [10, 12–16]. Understanding women’s perspectives on contraception following genital fistula repair and correcting misinformation is important for protecting women’s post-repair health.

Due to persistent challenges in ensuring access to emergency obstetric care, Uganda has a high prevalence of female genital fistula, with approximately 2% of women reporting history of fistula symptoms in nationally representative surveys of reproductive-aged women [17]. Stillbirth is a common consequence of childbirth resulting in fistula formation, and Ugandan society places high social value on large families, with total fertility rate of 5.4 children [17]. This combination of higher fertility norms and infant loss may limit both knowledge and interest in contraception; however, there is not yet data on post-repair contraceptive adoption among this population. Research from other sub-Saharan African countries is heavily quantitative and few studies followed women longitudinally, limiting our understanding of women’s unique decisions and experiences of a complex issue [10, 12–16].

To inform patient-centered contraceptive counseling among Ugandan women who have undergone genital fistula repair, we aimed to understand current practices, preferences, and unmet needs among a cohort of Ugandan women during the 12-month period following genital fistula repair, employing both quantitative and qualitative methods.

## Methods

This analysis was situated within a sequential explanatory mixed-methods study on recovery and reintegration following genital fistula repair surgery in Uganda, described in detail elsewhere [18]. Study participants were women who received genital fistula surgery at Mulago National Teaching and Referral Hospital in Kampala, Uganda. Fistula repair is provided by the urogynaecology division as an ongoing surgical service and supplemented by four to five targeted fistula repair camps annually. This analysis is focused on contraception among women in the 12-months following genital fistula repair, including quantitative data regarding post-repair contraceptive behaviours collected quarterly and qualitative data reflecting women’s post-repair contraceptive preferences at 12-months following fistula repair.

### Quantitative component

We recruited 60 women at the time of genital fistula surgery for 12-month longitudinal cohort participation. Our study was launched in December 2014 and recruitment lasted through June 2015. Study follow-up was completed in August 2016. Women were eligible if they spoke Luganda or English, resided in a community with cellular telephone coverage, and were able to provide informed consent for study participation. Individuals under age 18 years who experience genital fistula are considered emancipated minors; thus, no age-related eligibility restrictions were defined. All eligible participants were enrolled in the study following the provision of informed consent. Quantitative data were captured at baseline, 3-, 6-, 9- and 12-months post-surgery. Questions included sociodemographic characteristics; incontinence severity (International Consultation on Incontinence Questionnaire, Short Form) [19], sexual activity, contraceptive use, and pregnancy intention and status.

### Qualitative component

A purposively selected sample of 30 women from the quantitative longitudinal cohort were invited by the study team for an in-depth interview following the conclusion of the 12-month quantitative portion of the study to supplement our quantitative findings. Interviews took place from January-August 2016. Women were selected for a range of physical and psychosocial recovery experiences based on our quantitative data. In-depth interviews were conducted in-person in a private room at Mulago Hospital and lasted approximately 1–1.5 h each. The in-depth interview guide included open-ended questions on women’s post-repair recovery and reintegration experiences, including their relationships with their partners, sexual experiences, reproductive intentions, and contraceptive attitudes and use. Interviews were translated and transcribed into English for analysis.

### Analysis

Quantitative data analyses were performed utilising Stata v17 software (StataCorp, College Station, TX, USA). Univariate analyses were performed to describe participant characteristics. Descriptive statistics, including the mean and standard deviation for continuous variables and the number and proportion for categorical variables, were utilised to describe relationship status, fertility status, and contraceptive use among the longitudinal cohort over the 12-month follow-up period. We compared sexual activity and contraceptive use at 12 months by age group, parity, relationship status, educational attainment, number of living children, household asset score, time lived with fistula, urinary incontinence, and whether or not they were trying for pregnancy using Fisher’s exact test. Analyses of contraceptive use at 12 months included only those individuals reporting current sexual activity and included the 2 individuals who had reported sterilization on baseline questionnaire. These analyses used compressed categories for certain variables based on sample size. Qualitative data analyses were performed utilising Atlas.ti software. Transcripts from in-depth interviews were coded using inductive and deductive codes within Atlas.ti software, which were analysed to understand women’s sexual behaviours, experiences, and contraceptive use following recovery from obstetric fistula surgery. Two members of the research team were involved in coding the qualitative data (HN and AE), one Ugandan and one American. Coding disagreements were resolved by discussion. Coded data were analysed thematically to describe the different dimensions and commonalities of each theme and the patterns and linkages between themes.

### Ethical approval

The study protocol was approved by the Makerere University School of Medicine Research and Ethics Committee (Ref# 2014-052) and the University of California, San Francisco Human Research Protection Program, Committee on Human Research (IRB# 12-09573 and IRB# 15-17467) and the Uganda National Council for Science and Technology (REF#:1541212101). All individuals eligible for participation underwent an informed consent process; those individuals unable to provide signatures for informed consent provided thumbprint confirmation. All study methods were performed in accordance with the relevant guidelines and regulations.

## Results

### Sociodemographic characteristics

The median age was 28 years (interquartile range [IQR]: 21–36 years) for participants in the longitudinal study and 31.5 years (IQR: 27–38 years) for participants in the nested qualitative study (Table 1). Almost half of the participants (48% and 50%, respectively) were either married or living together with their partners. Many were separated from their partners or divorced (27% and 37%, respectively). Most of the participants (68% and 63%, respectively) had some primary education, whereas few had secondary education. Husbands were the main financial supporters of participants (40% and 43%, respectively), followed by themselves (28% and 33%, respectively), and relatives (32% and 23%, respectively). Many households had a mobile phone (65% and 57%, respectively), electricity (43% and 65%, respectively) or owned some land (47% and 37%, respectively). Most women had vesicovaginal fistula (98%); one woman had rectovaginal fistula and was included in our quantitative but not our qualitative sample (not shown).

**Table 1:** Sociodemographic characteristics of participants at study enrolment

	Longitudinal cohort		Nested qualitative cohort	
	N = 60		N = 30	
	N	%	N	%
Sociodemographic characteristics				
Age^a^, years	28 (21–36)		31.5 (27–38)	
Age at marriage^a^_,_ years	18 (17–20)		18.5 (17–21)	
Marital status				
Married	7	11.7	4	13.3
Living together	22	36.7	11	36.7
Divorced/separated	16	26.7	11	36.7
Widowed	3	5.0	0	0.0
Single/never married	12	20.0	4	13.3
Household characteristics				
Number of household residents^a^	4 (2–6)		4 (2–6)	
Other adult(s) in household	47	78.3	22	73.3
District of residence				
Central	57	95.0	31	99.7
Eastern	3	5.0	0	0.0
Socioeconomic status				
Educational attainment				
None	10	16.7	5	16.7
Some primary	24	40.0	10	33.3
Completed primary	17	28.3	9	30.0
Some secondary or more	9	15.0	6	20.0
Work outside of home	26	43.3	12	40.0
Primary source of financial support				
Self	17	28.3	10	33.3
Husband/partner	24	40.0	13	43.3
Relatives	19	31.7	7	23.3
Selected household assets				
Piped water	9	15.0	6	20.0
Electricity	26	43.3	39	65.0
Mobile phone	39	65.0	17	56.7
Land	28	46.7	11	36.7
Fistula characteristics				
Type of fistula				
VVF alone	21	75.0	19	70.4
RVF alone	1	3.6	0	0.0
VVF and RVF	2	7.1	2	7.4
Other	4	14.3	6	22.2
Length of time lived with fistula				
< 1 month	8	13.3	5	16.7
1–3 months	20	33.3	9	30
3–12 months	8	13.3	3	10
1–2 years	2	3.3	1	3.3
3–5 years	5	8.3	2	6.7
> 5 years	17	28.3	10	33.3
Repair outcome				
Successful	50	83.3	28	93.3
Unsuccessful	10	16.7	2	6.7

^a^Median (interquartile range); *N* number, *VVF* vesicovaginal fistula, *RVF* rectovaginal fistula

### Contraceptive counselling, resumption of sexual activity, and contraceptive use following surgery

Counselling on resuming sexual intercourse (97%) was routinely reported at the time of fistula surgery hospitalization, although fewer participants reported having received contraceptive counselling (59%). Women gradually resumed sexual activity following surgery, with 6.8% reporting sexual activity after 3 months, 32% after 6 months, and 50% reporting sexual activity after 12 months (Table 2). A total of six women became pregnant during the study period, including two within 3 months post-fistula repair. Across the 12-month follow-up, the large majority of sexually active women (83%) reported not trying for pregnancy. Despite this, contraceptive use was low but increased over the study follow-up, with 36% reporting method use at the time of fistula surgery compared with 75% at 12 months.^1^ At 12 months post-surgery, female sterilisation was the most common method reported among contraceptive users (38%), followed by oral contraceptive pills (13%), implants (8%), intrauterine devices (IUDs; 4%) and injections (4%). Condom use was rare. No participants reported use of traditional methods.

**Table 2:** Sexual activity and family planning use among study participants over the 12 months of follow-up after obstetric fistula surgery

	Baseline		3 months		6 months		9 months		12 months	
	N = 60		N = 59		N = 55		N = 55		N = 58	
	N	%	N	%	N	%	N	%	N	%
Partnered	30	50.0	25	42.4	28	49.1	28	50.9	30	51.7
Sexually active	11	18.3	4	6.8	18	31.6	26	47.3	29	50.0
Pregnancy status^a^	*N* = *11*		*N* = *4*		*N* = *18*		*N* = *26*		*N* = *29*	
Pregnant	0	0.0	2	50.0^b^	0	0.0	0	0.0	4	13.8
Trying for pregnancy	0	0.0	0	0.0	2	12.5	4	15.4	1	3.5
Not trying for pregnancy	11	100.0	2	50.0	16	87.5	22	84.6	24	82.8
Contraception^c^	*N* = *11*		*N* = *2*		*N* = *16*		*N* = *22*		*N* = *24*	
None	7	63.6	0	0.0	9	56.3	13	59.1	6	25.0
Condom	1	9.1	0	0.0	0	0.0	2	9.1	0	0.0
Oral contraceptives	0	0.0	0	0.0	0	0.0	0	0.0	3	12.5
IUD	1	9.1	0	0.0	0	0.0	1	4.6	1	4.2
Implant	0	0.0	0	0.0	1	6.3	0	0.0	1	4.2
Injection	0	0.0	0	0.0	1	6.3	1	4.6	3	12.5
Sterilisation^c^	2	18.2	2	100.0	5	31.3	5	22.7	9	37.5

^a^Among those sexually active

^b^Both participants reporting pregnancy at 3 months post-surgery experienced spontaneous abortion

^c^Among those responding not pregnant or not trying for pregnancy; IUD, intrauterine device

^d^Individuals reporting sterilization prior to fistula surgery (n = 2) are included within the contraceptive use distribution at all follow-up time points

The sociodemographic and clinical characteristics associated with sexual activity at 12-month follow up included relationship status and urinary incontinence (Table 3). Women whose urinary incontinence was resolved were also more likely to report resumption of sexual activity than those without incontinence (62% vs. 26%, p = 0.024). Women married or living with partners were also more likely to report sexual activity than those without partners (80% vs. 18%, p < 0.001). Women with 1 or more living children were more likely to report resumption of sexual activity than women with no children, slightly short of significance (61% vs. 30%, p = 0.052). However, marital status also was patterned by parity, with 45% of women with no children currently married compared to 55% of women with children (p = 0.582).

**Table 3:** Relationships between sociodemographic characteristics, selected fistula characteristics, and sexual activity or contraceptive use at 12 months

	N	Sexually active at 12 m					Contraceptive use at 12 m^*c*^				
		Yes		No		p-value	Yes		No		p-value
		N = 29		N = 29			N = 18		N = 11		
		N	%	N	%		N	%	N	%	
Age group, years						0.382					0.078
< 20	10	4	40.0	6	60.0		1	25.0	3	75.0	
20–29	22	12	54.6	10	45.5		6	50.0	6	50.0	
30–39	16	10	62.5	6	37.5		9	90.0	1	10.0	
≥ 40	10	3	30.0	7	70.0		2	66.7	1	33.3	
Number of children						0.057					0.012
None	20	6	30.0	14	70.0		1	16.7	5	83.3	
1–3	24	13	54.2	11	45.8		8	61.5	5	38.5	
4 +	14	10	71.4	4	28.6		9	90.0	1	11.1	
Relationship status^b^						< 0.001					1.000
Married or living together	30	24	80.0	6	29.0		3	60.0	2	40.0	
Separated, widowed, single	28	5	17.9	23	82.1		15	62.5	9	37.5	
Completed primary education						0.792					0.001
No	32	17	53.1	15	46.9		15	88.2	2	11.8	
Yes	26	12	46.2	14	53.9		3	25.0	9	75.0	
Household asset ownership^a^						0.590					0.948
≤ 1	15	6	40.0	9	60.0		4	66.7	2	33.3	
2	13	6	46.2	7	53.9		3	50	3	50.0	
3	11	5	45.5	6	54.6		3	60	2	40.0	
4 or more	19	12	63.2	7	36.8		8	66.7	4	33.3	
Time lived with fistula						0.221					0.653
< 1 m	8	6	75.0	2	25.0		4	66.7	2	33.3	
1–3 m	20	12	60.0	8	40.0		7	58.3	5	41.7	
3–12 m	7	3	42.9	4	57.1		3	100.0	0	0.0	
1–5 y	6	3	50.0	3	50.0		1	33.3	2	66.7	
> 5 y	17	5	29.4	12	70.6		3	60.0	2	40.0	
Urinary incontinence^b^						0.024					1.000
Yes	19	5	26.3	14	73.7		3	60.0	2	40.0	
No	39	24	61.5	15	38.5		15	62.5	9	37.5	
Trying for pregnancy											0.379
Yes							0	0	1	100	
No							18	64.3	10	35.7	

^a^Household assets included piped water, radio, bicycle, flush/pour flush toilet, television, refrigerator, electricity, mobile phone, land

^b^At the time of follow-up data collection (i.e., 12 months post-surgery)

^c^Denominator includes only those individuals reporting sexual activity

Among a reduced sample of sexually active individuals at 12 months (n = 29), number of living children and completion of primary education were both significantly associated with contraceptive use (Table 3). Contraceptive use varied by the number of living children a woman had; 90% of women with 4 or more children were more likely to report contraceptive use compared to 62% of women with 1–3 children and 17% of those with no children (p < 0.012). Contraceptive use was lower among women who had completed primary education (25%) compared to those with less than primary education (88%, p < 0.001); however, education and parity were related, with 50% of women who had completed primary education reporting 1 or more living children compared to 76% of women who had not (p = 0.055, not shown).

### Fertility preferences

Fertility preferences varied across the qualitative sample, with some women sharing a desire to become pregnant immediately, others wanting to postpone until later, and some not interested at all in another birth. Qualitative participants desiring to have children immediately were generally younger, had fewer children, and had experienced stillbirth at fistula development. One 23-year-old participant who lost her only child when she developed a fistula could not wait to have a child; in few words she asserted, *‘Right* *now* *I* *want* *a* *baby.’* *(Interviewee,* *23* *years* *old,* *no* *living* *children)* Other participants reported being anxious to become pregnant immediately due to pressure from partners who were concerned about infertility, some with the added competition from co-wives. One 24-year-old participant whose husband desperately wanted her to have a girl child shared*:*

*I* *was* *so* *anxiously* *waiting* *for* *[pregnancy].….* *Well* *after* *the* *six* *months,* *it* *was* *the* *only* *thing* *which* *I* *was* *waiting* *for….* *T*he man was also suspicious; he always said, “You see some people whose uteruses are taken out are sometimes unaware of it. So maybe you are just unaware *[*that yours was removed and you *cannot* *conceive].* *(Interviewee,* *24* *years* *old)*

Few participants overall in the quantitative sample reported trying for pregnancy during the study period (n = 7) but a couple of young women (median age 22) began trying for pregnancy starting at 6 months post-surgery (Table 2).

Individuals who shared that they wanted to have a child, or more children, planned for both the short and longer term, with some wanting to become pregnant in the next 1–2  years, and a few younger women wanting to wait further out. Women had various reasons for waiting. Some wanted to ensure that they were fully healed from fistula, others wanted to take some time to work and improve their economic status before the next child, and others felt aggrieved by prior low-quality relationships and were concerned about making sure they would find new caring and supportive partners.

*When* *I* *look* *at* *my* *neighbours* *or* *friends’* *children,* *then* *I* *feel* *I* *need* *a* *child* *but* *truthfully* *speaking,* *I* *am* *only* *planning* *to* *give* *birth* *when* *I* *have* *completely* *healed.* *(Interviewee,* *41* *years* *old)*

*I* *think* *about* *remarrying* *to* *someone* *who* *already* *has* *a* *home* *but* *not* *renting.* *In* *fact,* *I* *don’t* *want* *a* *man* *to* *marry* *me* *from* *a* *rental* *house* *(jeers* *in* *disgust).* *After* *these* *young* *men* *have* *impregnated* *you,* *they* *desert* *you* *in* *the* *(single* *roomed)* *rental* *house* *and* *then* *you* *suffer* *with* *both* *the* *child* *and* *paying* *rent;* *I* *am* *not* *interested* *in* *such….* *[If* *I* *get]* *the* *right* *person,* *someone* *sent* *from* *God,* *someone* *who* *even* *has* *some* *money,* *I* *could* *hope* *of* *giving* *birth* *to* *maybe* *four* *children;* *those* *would* *be* *enough* *for* *me.* *(Interviewee,* *32* *years)*

The number of children desired varied across participants who expressed interest, with most wanting several children. Those participants who reported not wanting any more children all had at least one living child, with those who had undergone sterilization having a minimum of three living children. These participants were in their late thirties and forties and felt they had had moved beyond their childbearing years.

*I* *am* *old* *now,* *why* *would* *I* *give* *birth?* *In* *fact,* *I* *fear* *now.* *With* *where* *I* *have* *reached* *so* *far,* *my* *only* *wish* *is* *to* *heal* *and* *I* *don’t* *think* *about* *childbirth.* *(Interviewee,* *40* *years* *old,* *lived* *with* *fistula* *for* *22* *years)*

*I* *can’t* *imagine* *people* *seeing* *me* *pregnant* *at* *this* *age.* *My* *kid* *goes* *into* *labour* *and* *I* *also* *follow?* *That* *would* *mean* *that* *I* *am* *not* *well* *upstairs.* *That* *can’t* *be.* *(Interviewee,* *48* *years* *old)*

The few younger women who didn’t want additional children highlighted their focus on working to care for and educate the children that they already had. Some mentioned the intersecting fear of not being able to provide for their current children if they were to develop fistula again from a subsequent childbirth.

#### Desire for contraception

Participant narratives regarding contraception mirrored our findings around fertility preferences, with individuals thoughtfully choosing to prevent pregnancy for reasons related to both their fistula experiences and broader life circumstances. Fistula-related influences included the desire to fully recovery from the fistula experience and avoid future adverse outcomes, as well as the desire to enjoy life again after suffering. Other reasons included a general desire to space children and concerns about partner commitment.

### Desire to fully recover from fistula repair or prevent fistula repair breakdown

Some participants noted that they quickly adopted contraception to avoid becoming pregnant before they were fully recovered from the fistula repair. Some participants expressed their willingness to abstain from sex for longer periods of time, whereas others abstained from sex for a short period but later adopted contraception, particularly those who indicated doubts regarding whether their partners would respect their wishes to delay intercourse.

*In* *that* *year,* *I* *refused* *to* *have* *intercourse* *with* *him* *because* *I* *felt* *that* *I* *hadn’t* *healed* *well…* *The* *reason* *why* *I* *had* *taken* *the* *injection* *that* *time* *was* *because* *I* *was* *afraid* *that* *he* *would* *force* *himself* *on* *me,* *something* *which* *I* *didn’t* *want.* *So* I chose to have that injection. *(Interviewee,* *23* *years* *old)*

One woman who had left her partner just prior to her fistula surgery due to an abusive relationship indicated no desire to find a new partner until she had healed:

*I* *cannot* *get* *a* *man* *before* *getting* *better.* *I* *would* *love* *to* *take* *three* *more* *years* *(without* *having* *sex)* *and* *then* *get* *a* *man.* It’s what my heart wants, and that’s after getting much better. *(Interviewee*, *29* *years* *old)*

Other participants were motivated to adopt contraceptives to avoid pregnancy and the risk of fistula recurrence because of the negative fistula experience. One interviewee observed, *‘I* *have* *to* *keep* *away* *from* *giving* *birth* *because* *it* *was* *through* *it* *that* *I* *got* *the* *problem* *(fistula)*.’ *(Interviewee,* *32* *years* *old).*

Finally, some participants had been specifically counselled by health workers about contraception because of existing complications and the risks they would be exposed to if they conceived. One participant explained, ‘*The* *doctors* *did* *that* *(placing* *an* *IUD)* *because* *of* *the* *way* *how* *I* *had* *got* *torn* *and* *they* *said* *that* *would* *help* *me* *for* *three* *years* *without* *giving* *birth.*’ *(Interviewee,* *32* *years* *old).* Although this participant did not have a partner at the time of the interview, she received an IUD because she was at high risk of fistula recurrence and indicated a desire to focus on her business and educating her existing children.

### History of unplanned pregnancy or poor obstetric experiences

A history of unplanned pregnancies and certain obstetric events, such as multiple caesarean sections, compelled some participants to consider contraception to avoid reoccurrence. This perspective was voiced by one interviewee who underwent multiple caesarean surgeries and fistula repairs and wanted to avoid additional surgeries:

But since this [last] *time* *I* *got* *pregnant* *without* *having* *prepared* *for* *it,* *then* *I* *think* *I* *need* *to* *go* *for* *family* *planning* *as* *well.* *I* *need* *[contraception*] because *[the* *doctors]* have always been operating me for *my* *births.* *T*hey have so far operated me on for *four* *kids* *and* *then* *the* *bladder,* *it* *has* been seven times of operation. All in all, they have operated me *11* times *and* *it* *is* *not* *safe* *for* *my* *body* *for* *all* *those* *times.* *I* *don’t* *want* *to* *go* *back* *to* *the* *theatre.* *(Interviewee*, *40* *years* *old)*

### Desire to end childbearing or space children

Several participants indicated not wanting to conceive again and adopted contraceptives, including permanent methods, particularly those with multiple children. Other participants shared that they adopted contraceptives to space their children rather than prevent all future pregnancies: ‘*I* *would* *like* *[family* *planning]* *if* *I* *give* *birth* *frequently* *…* *because* *I* *would* *like* *my* *child* *to* *grow* *up* *to* *a* *better* *stage* *….* *But* *if* *not,* *I* *cannot* *use* *it*.’ *(Interviewee,* *29* *years* *old).*

### Perceived partner commitment to the relationship

Fistula can impact relationships with partners, and some participants experienced volatility in their intimate relationships. Although most quantitative participants (80%) reported having been married, only 60% of these were married or living with a partner at the time of the fistula surgery, and 33% reported having been divorced. At the 12-month follow-up, only 52% reported currently being married or living with a partner. The perceived stability of their intimate relationships affected the women’s views on contraception, and some participants were not willing to have children in relationships they considered to be temporary, especially in cases in which their partners were not financially supportive; thus, they indicated a willingness to use contraception to avoid becoming pregnant with such a partner:

*It* *also* *depends* *on* *which* *kind* *of* *partner* *you* *have.* *Currently,* *if* *I* *met* *a* *person* *who* *would* *only* *give* *me* *money* *for* *food* *or* *pay* *rent,* *then* *I* *know* *that* *such* *a* *person* *is* *temporary* *and,* *therefore,* it’s not a good idea to have a child for such a person. *(Interviewee,* *19* *years* *old)*

### Desire to enjoy life after fistula

A desire to enjoy sexual life after fistula repair, following suffering with fistula, motivated some participants to adopt contraception, although they did not always involve their partners in this decision for fear of pressure to have children. One participant who finally felt free to enjoy her life after recovering from fistula stated that she preferred her partner to think her uterus was removed rather than that she was using a contraceptive: **‘***These* *days* *it* *is* *all* *about* *enjoying* *(sex)* *and* *eating* *money.*^2^*You* *just* *try* *to* *find* *all* *means* *of* *telling* *him* *that* *the* *uterus* *was* *removed.* *(Interviewee,* *32* *years* *old).*

### Contraceptive preferences

Several participants indicated they had completed childbearing and sought permanent birth control. Even though only 4% of participants in full sample relied on injections, the participants in the nested qualitative cohort consistently expressed a preference for the contraceptive injection. Several participants sharing this would be new contraceptive injection users, while others had used it in the past. Some identified family members or friends as users. Fewer participants shared the oral contraceptive pill as their preferred method.

#### Barriers to contraception

Although some women reported using contraception upon the resumption of sexual activity post-fistula surgery, significant unmet need was identified among our longitudinal cohort during the 12 months following fistula surgery (Table 2). To fully understand variations in contraceptive uptake, participants in the qualitative arm who reported not adopting contraception during the year following fistula surgery despite a desire to avoid pregnancy were requested to share their decision-making process. The reasons cited included perceptions of low fecundity and fears and misconceptions about contraceptives. Others reported they were advised by health care workers to stop using contraception or that they received unclear or worrisome information from health providers.

### Perceptions of not being at risk of pregnancy

Several participants felt that they were not at risk of pregnancy due to previous failure to conceive or altered menstruation, including amenorrhea. Women who considered themselves infertile felt no need to use contraceptives:

*T*here is no need for me using [family planning] *since* *I* *don’t* *give* *birth,* *so* *why* *should* *I* *use* *them?* *That’s* *what* *I* *think* *since* *it’s* *been* *a* *long* *time*…. I don’t know whether it is a God-made form of family *planning,* but ever since I got that pregnancy (that resulted *in* *the* *fistula)* *and* *the* *second* *one* *which* *was* *terminated,* *I* *have* *never* *gotten* *another* *one,* *and* *yet* *I* *have* *never* *used* *any* *family* *planning* *medicine.* *(Interviewee,* *28* *years* *old)*

Similarly, women who experienced altered menstruation or amenorrhea felt these symptoms were indicative of infertility and were not using contraception. One woman described her menstrual pattern:

*I* *don’t* *bleed* *much* *blood;* *I* *get* *my* *period* *for* *a* *small* *time,* *like* *for* *one* *or* *two* *days,* *and* *then* *it* *stops…* *since* *the* *removal* *of* *that* *baby.* *I* *would* *sometimes* *miss* *the* *periods* *for* *two* *months* *and* *then* *they* *reappear* *in* *the* *third* *month.* (Interviewee, 39 years old)

Another participant shared*,* *‘I* *cannot* *plan* *when* *to* *have* *babies* *because* *I* *don’t* *get* *my* *periods.* *From* *the* *time* *I* *had* *an* *operation* *for* *the* *delivery* *of* *my* *child,* *I* *didn’t* *have* *my* *periods* *again’* (Interviewee, 22 years old).

One participant discussed how she planned to start contraception after resuming her periods and had assumed that she was infertile due to amenorrhea but was surprised by an unintended pregnancy: ‘*I* *was* *waiting* *to* *first* *get* *my* *periods,* *but* *by* *the* *time* *I* *got* *them,* *I* *went* *to* *hospital* *for* *a* *check-up* *and* *the* *test* *came* *out* *positive;* *I* *was* *pregnant,’* (Interviewee, 20 years old).

### Fears or misconceptions about contraception including infertility

Participants revealed a variety of fears of infertility and misconceptions regarding contraceptives. Several expressed concerns about the effects that contraceptive methods might have on their future fertility: ‘*I* *have* *never* *used* *[family* *planning],* *and* *it* *is* *not* *good* *because* *you* *could* *reach* *a* *time* *of* *desiring* *to* *give* *birth* *and* *you* *fail* *to* *get* *pregnant.’* (Interviewee, 28 years old).

Some participants shared concerns about particular methods that they feared would cause harm. Several thought that contraceptives would give them fibroids. They also discussed fears of cancer due to both hormonal and non-hormonal methods, including condoms. Concerns that contraception did not actually work to prevent pregnancy also were expressed. One participant stopped taking oral contraceptive pills due to information about their effectiveness that she received from others, ‘*I* *was* *told* *that* *those* *pills* *are* *not* *reliable* *and* *that* *I* *should* *stop* *using* *them….* *So,* *I* *stopped* *taking* *them.*’ (Interviewee, 19 years old). These concerns were primarily derived from information from people in their communities:

Well sometimes you might try and swallow [oral contraceptive pills] but they say that tablets cause fibroids, except these things they put under the arm’s skin (implant). (Interviewee, 23 years old)

Family planning? I don’t like family planning. People say that it is bad; it causes fibroids. Every time, I hear women having issues in their menstrual periods, eh! People speak ill about family planning; they say that it is bad…people say things like, *‘I* *used* *family* *planning* *and* *it* *caused* *me* *some* *issues;* *I* *don’t* *stop* *bleeding* *when* *I* *am* *in* *my* *periods’* or *‘I* *get* *fibroids,* *which* *I* *never* *had* *before.’* *And* *besides,* *the* *condoms* *that* *they* *recommend* *also* *cause* *illnesses.* *They* *cause* *cancer;* *they* *are* *the* *main* *causes* *of* *cancer* *in* *people,* *including* *those* *family* *planning* *tablets.* *In* *fact,* *I* *have* *never* *swallowed* *them.* (Interviewee, 32-years old)

### Low knowledge about contraception

At times, however, participants cited health personnel as information sources, which may indicate miscommunication or misinformation, or at least the need for more comprehensive counselling on preferred methods:

*The* [health workers] *said* *that* *for* *the* *injections,* *they* *reduce* *the* *ova* *that* *someone* *has* *and* *then* *the* *capsule* *(implant),* *it* *brings* *about* *over* *bleeding* *and* *having* *some* *complications.* (Interviewee, 28 years old)

Other deterrents included a lack of knowledge regarding available methods among some participants. One young participant who developed fistula at her first pregnancy stated, *‘I* *have* *never* *used* *family* *planning,* *[and]* *am* *actually* *ignorant* *about* *it.*’ (Interviewee, 22 years old).

### Religious beliefs

Religious beliefs were also reported as deterrents to contraceptive use among some participants, particularly those who identified as born-again who felt that their family sizes would entirely depend on the will of God. One participant noted**, ‘***I* *don’t* *like* *[family* *planning].* *It* *is* *only* *God* *that* *can* *decide* *for* *me* *on* *that.* *I* *just* *fear* *it.* *[Our* *religion* *tells]* *us* *not* *to* *go* *for* *family* *planning.’* (Interviewee, 22 years old).

## Discussion

Our study found that despite increasing contraceptive adoption over the year following genital fistula repair among Ugandan women, unmet need for contraception was persistent. Provider recommendations are generally that pregnancy be avoided for somewhere between 3 and 12 months following fistula repair, however, in our cohort several participants experienced another pregnancy before that time. Participant narratives revealed that the factors facilitating and impeding post-repair contraceptive adoption occurred at individual, interpersonal and community levels, framing key domains for protecting reproductive health and advancing reproductive empowerment among this population.

We observed an increasing trend in contraceptive initiation among our study participants over time following fistula surgery, from 36% at 6 months to 73% at 12 months. In this study, most women resumed sexual activity between 6 and 9 months following surgery. Many women who were sexually active and who did not desire pregnancy reported not using contraception. A relatively high proportion of women (40%) reported not being counselled on contraception at the time of fistula surgery. Our findings on the lack of knowledge and access to preferred methods revealed a need for high quality contraceptive counselling including a range of methods.

Fertility preferences post-fistula repair vary across the reported literature, with many women reporting a desire to become pregnant following fistula repair [6]. In this study we observed a higher desire to become pregnant rapidly among younger women, especially those with no living children, even before the recommended one year after fistula surgery. This may be attributed to the natural desire to replace a child lost with another or by individual or societal expectations for childbearing, particularly given prevailing fertility rates in Uganda [17]. In a descriptive study of Malawian women undergoing fistula repair, 20% had a post-repair pregnancy with a median time to conception of 1.1 years (IQR 0.7–1.32 years) [12]. Some studies have identified desire for childbearing soon after fistula repair where there is no living child [14]. However, other studies have identified decreased interest in childbearing following fistula, as in a Congo study where most (86%) reported a decreased desire to have children after developing fistula [8].

The findings from this study identified some key challenges to reproductive empowerment among our study population occurring within individual, interpersonal, and health systems domains [20]. At the individual level, limited knowledge and appraisal were key barriers seen through low perceived risk of pregnancy, fears and misconceptions regarding contraceptives, lack of knowledge regarding contraceptive options, and religious beliefs. These findings suggest an immediate need for improving the quality of information provided during the counselling sessions following fistula repair in addition to broader educational efforts.

Furthermore, our findings on participant preferences suggest that contraceptive counselling should be expanded to acknowledge and provide the methods that women prefer. Knowledge empowerment and increased access to preferred contraceptives represent important and initial steps that can be taken to help Ugandan women follow post-fistula care recommendations, such as abstinence from sexual activity for 3–6 months and delayed childbirth for 1-year post-repair, which can help them attain their desired goals of having healthy children after fistula repair without fistula recurrence. In other countries, such as the Democratic Republic of the Congo, Malawi and Nigeria, studies have revealed a moderate knowledge of contraceptive methods among about 60% of surveyed women [13, 15, 16], prior to any intervention. However, contraceptive counselling can result in substantial increases, such as to 98% after intervention In the DRC [15]. Among other studies in sub-Saharan Africa, only 53%–61% of women reported being aware of available contraceptive methods at the time of study enrolment or the importance of preventing unintended consequences due to subsequent pregnancies [8–10]. Our study is limited by the lack of assessment of contraceptive method awareness among study participants as well as local contraceptive method availability, a systems-level determinant; however, improving contraceptive knowledge and increasing availability has been an ongoing goal of Ugandan government and partnering programs [21].

At the interpersonal level, partner influence is acknowledged as an important factor in women’s pregnancy decision-making, including within the contraceptive research from Uganda [22]. In this study, some women reported substantial pressure from their partners, and for our research participants, this experience was embedded within fears of infertility, infertility stigma, and the associated relationship consequences. The importance of partner influence has also been identified in Nigeria where partner disapproval of contraceptive use accounted for about one-third of non-adoption [14]. These findings emphasize the importance of engaging not only the women themselves, but also their partners in post-repair contraceptive counselling to optimize informed decision-making on post-repair contraception.

The abundance of myths and misconceptions regarding contraception among our study participants is also consistent with research in some other sub-Saharan African settings. In a Nigerian study, low uptake of post-fistula contraception (37%) was attributed to low socioeconomic status, culture, religion, and myths regarding contraception [13]. Some of these misconceptions are due to social norms within the communities to which these women belong, which often reflect more rural settings with high poverty. Tailoring of community awareness programmes may be necessary to increase knowledge not only among fistula survivors, but also within their communities to limit the influence of misconceptions and increase community support. Further support for the need for contraceptive education is evident in the limited range of method options discussed by our study participants, and from the literature which suggests geographic differences in unmet need for contraception across the country [23, 24]. Although it is a commonly cited barrier in studies regarding unmet need for contraceptives, [25, 26] access issues did not arise frequently in our study and we did not probe about it which represents a limitation to the current study. High quality post-surgical and contraceptive education for women who have undergone fistula repair, with linkages to access, may well contribute to improved health outcomes.

Some respondents in the qualitative arm of the study expressed their desires to delay or end childbearing, either to recover from the physical and psychological trauma experienced with fistula or due to a lack of desire for more children, particularly among those who already had at least one child. Others cited poor obstetric histories or the lack of supportive partners. In this study, 73% of participants adopted at least one contraceptive method within 12 months after fistula repair, with the largest proportion of respondents reporting the use of oral contraceptives or sterilisation compared with other methods (e.g., the injection, condoms, IUDs, and implants). Our quantitative findings on method used contrasted with the preferences voiced by our in-depth interview participants for the contraceptive injection. Sterilisation was more common among our study population. This may be due to the difficulties experienced during childbirth that resulted in fistula development and may have warranted permanent procedures, such as total hysterectomies, to save the woman’s life, particularly for those who had undergone sterilization prior to study entry. Abstinence was also reported as an adopted method by some respondents. Method mix reported by our participants is distinct from a cross-sectional study performed among women attending two obstetric fistula units in different Nigerian states for which the injection and implants were reported as the most common contraceptive methods [14], and from a nationally-representative Ugandan data which found injectables to be the most common method [27], suggesting geographical variability in preferences or availability.

Surprisingly, we found that contraceptive use in our study was inversely patterned by educational attainment, where women with higher educational attainment less likely to use contraception than women with lower educational attainment. However, our results suggest that these findings are an artifact of the small number of women with higher educational levels in our study, and the differences observed in the number of living children by women’s educational attainment and warrant further research in larger studies.

## Conclusions

Surgery to repair fistula is the first step in managing this condition. Holistic approaches to care include consideration for the physical, psychosocial, and economic needs of patients [28]. Our study demonstrates a discrepancy between the need and the utilization of post-surgical contraception, with half of women resuming sexual activity by 12 months. To achieve holistic care, recovery programmes must also consider the contraceptive needs of women post-repair.

Post-operative counselling at the time of fistula surgery should be reassessed to increase the number of women, and their partners when relevant, receiving high quality, patient-specific, contraceptive counselling. Existing health worker knowledge of contraceptive methods should also be reassessed periodically to ensure that patient-centred education, considering each patient’s concerns and preferences, is delivered before discharge.

## Data Availability

The datasets generated during and analysed during the current study are not publicly available due to limitations of the ethical approval involving the patient data and anonymity but are available from the corresponding author on reasonable request.

## Footnotes

1 Sensitivity analyses of contraceptive use across follow-up excluding those women reporting amenorrhea were 40% at the time of fistula surgery to 69% at 12 months. Overall contraceptive prevalence rate at 12 months includes 2 individuals who reported sterilization before fistula surgery.

2 The term ‘eating money’ refers to having fun with the partner when he is spending money in terms of entertainment or gifts.
